# A rare incidence of primary pulmonary undifferentiated pleomorphic sarcoma detected as an endobronchial mass and treated by bronchoscopic resection: a case report and literature review

**DOI:** 10.3389/fonc.2026.1746597

**Published:** 2026-05-08

**Authors:** Wei Wei, Huimin Wu, Hancheng Wang, Rongwen Ji, Zhe Sun, Songguo Li, Cong Liang, Ying Wang

**Affiliations:** 1 Department of Radiology, Anhui No.2 Provincial People’s Hospital, Hefei, Anhui, China; 2 Department of Pathology, Anhui No.2 Provincial People’s Hospital, Hefei, Anhui, China

**Keywords:** Bronchoscopy, case report, lung neoplasms, resection, sarcoma, tomography, x-ray computed, undifferentiated pleomorphic sarcoma

## Abstract

**Rationale:**

Primary pulmonary undifferentiated pleomorphic sarcoma (PPUPS) is a highly malignant tumor with extremely rare occurrence and is known for its distinct cellular pleomorphism. This tumor lacks specific differentiation markers. Consequently, PPUPS diagnosis requires thorough exclusion of other tumors through a comprehensive assessment of clinical, histopathological, and radiological outcomes.

**Patient concerns:**

A male patient of age 78 years presented to the hospital showing symptoms of continuous cough and blood-stained sputum for 1 month. Chest computed tomography detected a massive solid mass in the right lower lobe, stretching toward the right main bronchus. An endobronchial tumor blocking the right main bronchus was detected by bronchoscopy. We then conducted comprehensive histopathological and immunohistochemical analyses to elucidate the tumor’s characteristics.

**Diagnosis:**

On the basis of the findings of immunohistochemical and histopathological evaluations conducted for the specimen collected during bronchoscopic resection, the diagnosis of PPUPS was confirmed after other metastatic diseases and sarcomas were ruled out.

**Interventions:**

The patient declined further surgical treatment, radiotherapy, or chemotherapy, citing personal reasons and concerns.

**Outcomes:**

Owing to the increased surgical risk and the considerably reduced capacity of the patient’s lung, clinicians avoided conducting radical surgical resection; this decision was made after careful evaluation by multidisciplinary experts from the fields of respiratory medicine, anesthesiology, and thoracic surgery and consultation with the patient’s family. The patient survived for 38 months postoperation.

**Lessons:**

PPUPS can develop as a solitary endobronchial mass, appearing almost identical to primary bronchogenic carcinoma. For patients considered ineligible to receive radical surgery intervention, survival may be enhanced by promoting sustained local control through minimally invasive bronchoscopic resection. This approach confronts the established belief that the sole viable treatment option is aggressive surgical resection. Accurate diagnosis of PPUPS relies on high clinical suspicion and careful elimination of other sarcomas on the basis of immunohistochemical analysis findings.

## 1 Introduction

Undifferentiated pleomorphic sarcoma (UPS), designated earlier as malignant fibrous histiocytoma (MFH), has mesenchymal origin and mainly develops from the deep soft tissues of the extremities; this malignancy mostly occurs in the elderly population, particularly in patients aged 50–70 years (1). According to the WHO tumor categorization (2013 edition), MFH is not considered a distinct diagnostic entity; moreover, most of the corresponding lesions were reclassified as UPS (2). Primary pulmonary undifferentiated pleomorphic sarcoma (PPUPS), a highly uncommon subtype of primary pulmonary sarcomas (PPSs), shows poor prognosis and low patient survival (3). PPUPS has nonspecific clinical presentation and radiologic features; therefore, histopathological examination is critical for differential diagnosis between PPUPS and common primary lung epithelial tumors (4).

This article describes an extremely rare case of PPUPS that presented solely as an endobronchial lesion in the lower lobe segmental bronchus, but with no evidence of contiguous pulmonary parenchymal invasion. A thorough preoperative evaluation confirmed that the patient could not receive radical surgery. Subsequently, palliative endoscopic resection of the endobronchial tumor was achieved through flexible bronchoscopy with a polypectomy snare. The patient exhibited a favorable outcome, surviving at 38 months postoperation. This case shows the potential importance of utilizing local endoscopic therapy for some high-risk patients with PPUPS and supplements the scant literature on this rare tumor. The patient’s family provided written informed consent to document this case.

## 2 Case report

A male patient of age 78 years arrived to our hospital with following main complaints: productive cough, blood-streaked sputum, tightness in his chest, and exertional dyspnea. Before quitting smoking in 2020, he had a smoking history of 12 pack-year. At 1 month before admission, he began experiencing non-induced productive cough with thick sputum. He then sought treatment at a local clinic, where he was prescribed antitussive agents, expectorants, and oral antibiotics (specific medications are unclear due to unavailable records). However, his symptoms did not subside, and he still experienced intermittent episodes of blood-tinged sputum.

At 3 days prior to admission, he showed symptoms of dyspnea and tightness in the chest; these symptoms worsened with activity and alleviated with rest. The patient’s long-term hypertension was managed by oral nimodipine (1 tablet/day) despite irregular blood pressure monitoring. The patient also had a history of cerebral infarction, although specific details remain unclear. There was no history of hepatitis, tuberculosis, or malaria incidence.

The patient showed stable vital signs upon physical examination. Lung auscultation demonstrated diminished breath sounds in the right lower lung, absence of rales/rhonchi, and clear breath sounds in other fields. No other abnormalities were found.

**Figure 1:**
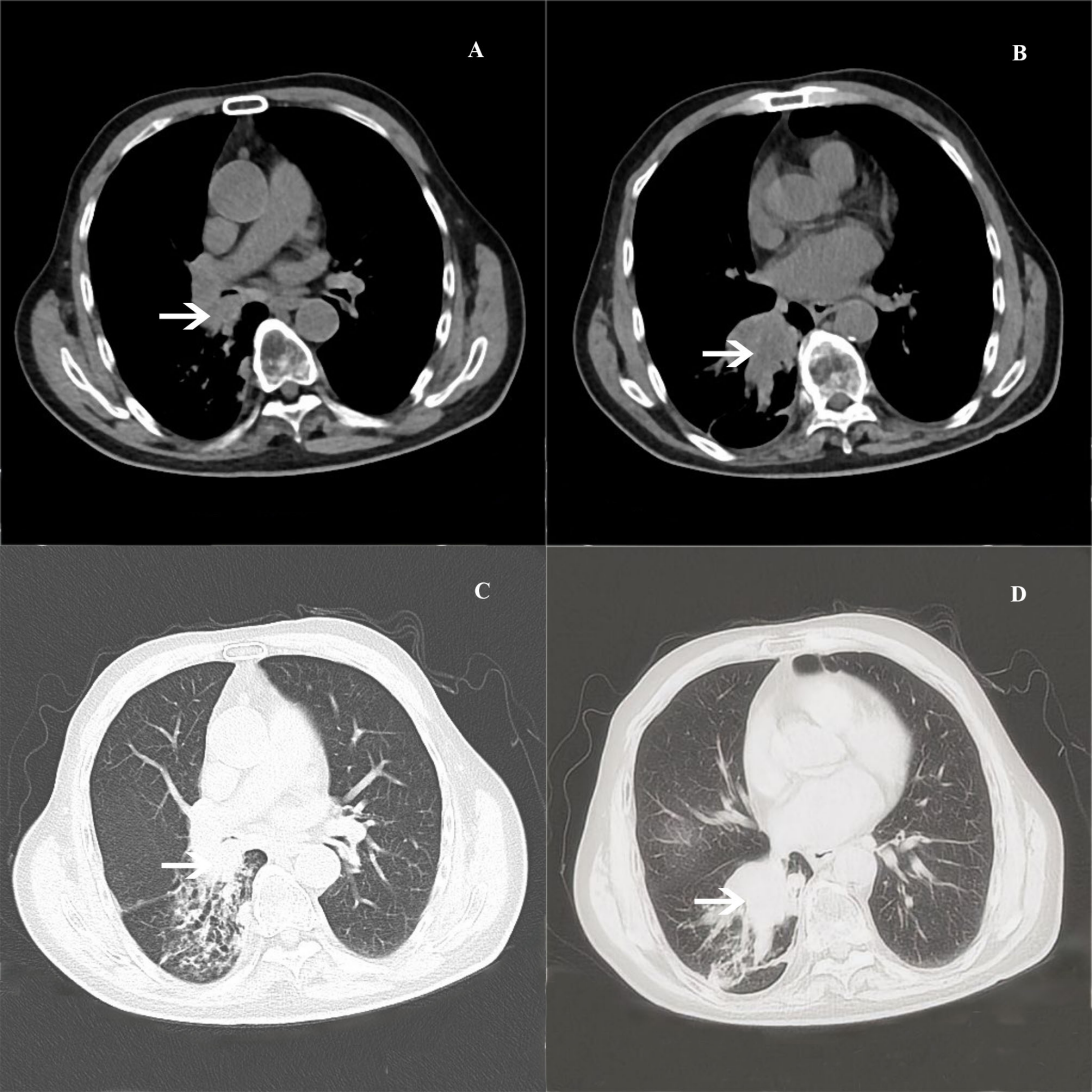
**(A–D).** Chest CT reveals a soft tissue mass (arrows) distributed along the right main bronchus as well as the right lower lobe bronchi of the lung.

**Figure 2:**
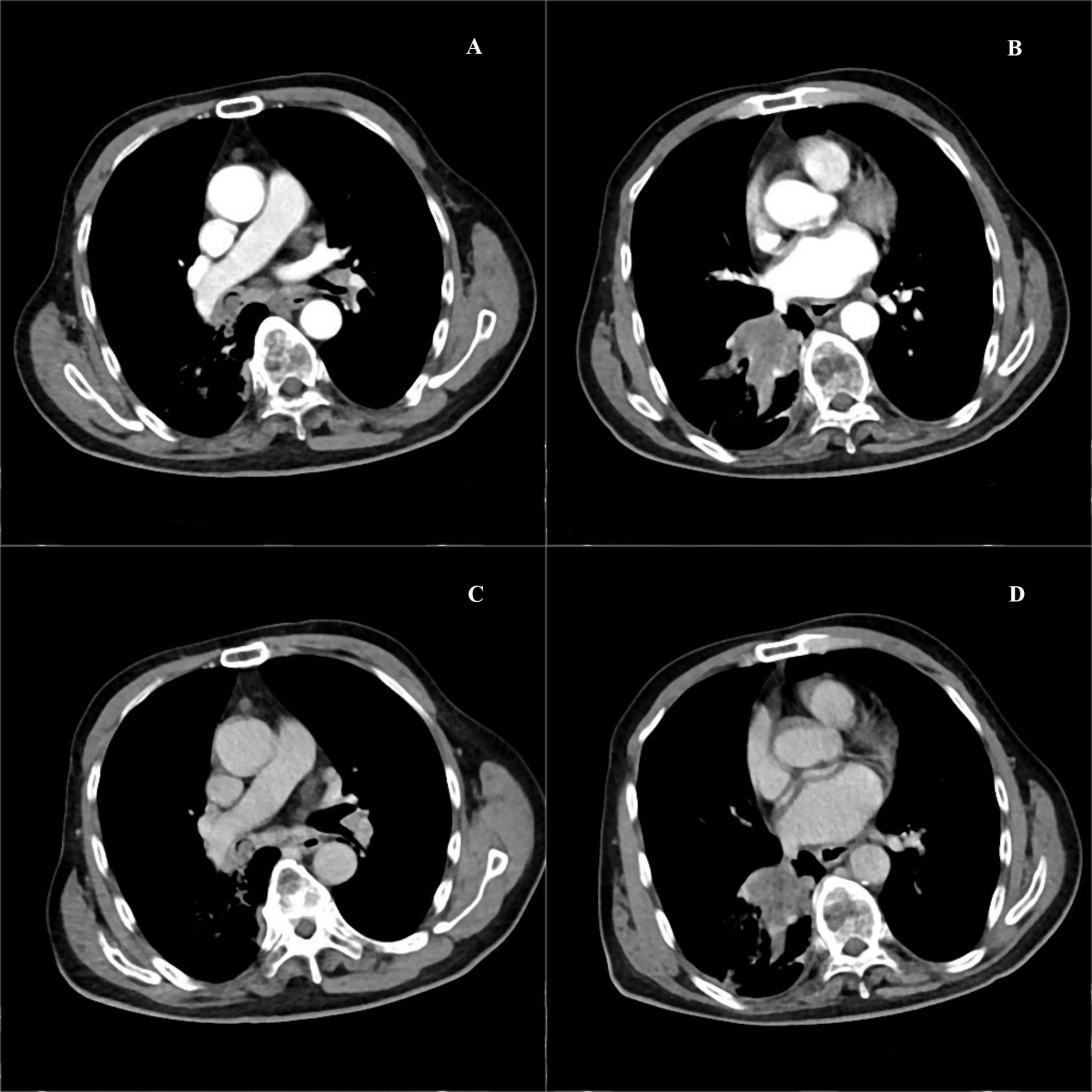
**(A–D).** Contrast-enhanced chest CT shows an irregular mass at the right lower hilum, extending along the right main bronchus and lower lobe bronchi. The mass displays indistinct boundaries and heterogeneous internal density. It shows progressively irregular enhancement following contrast agent administration.

**Figure 3:**
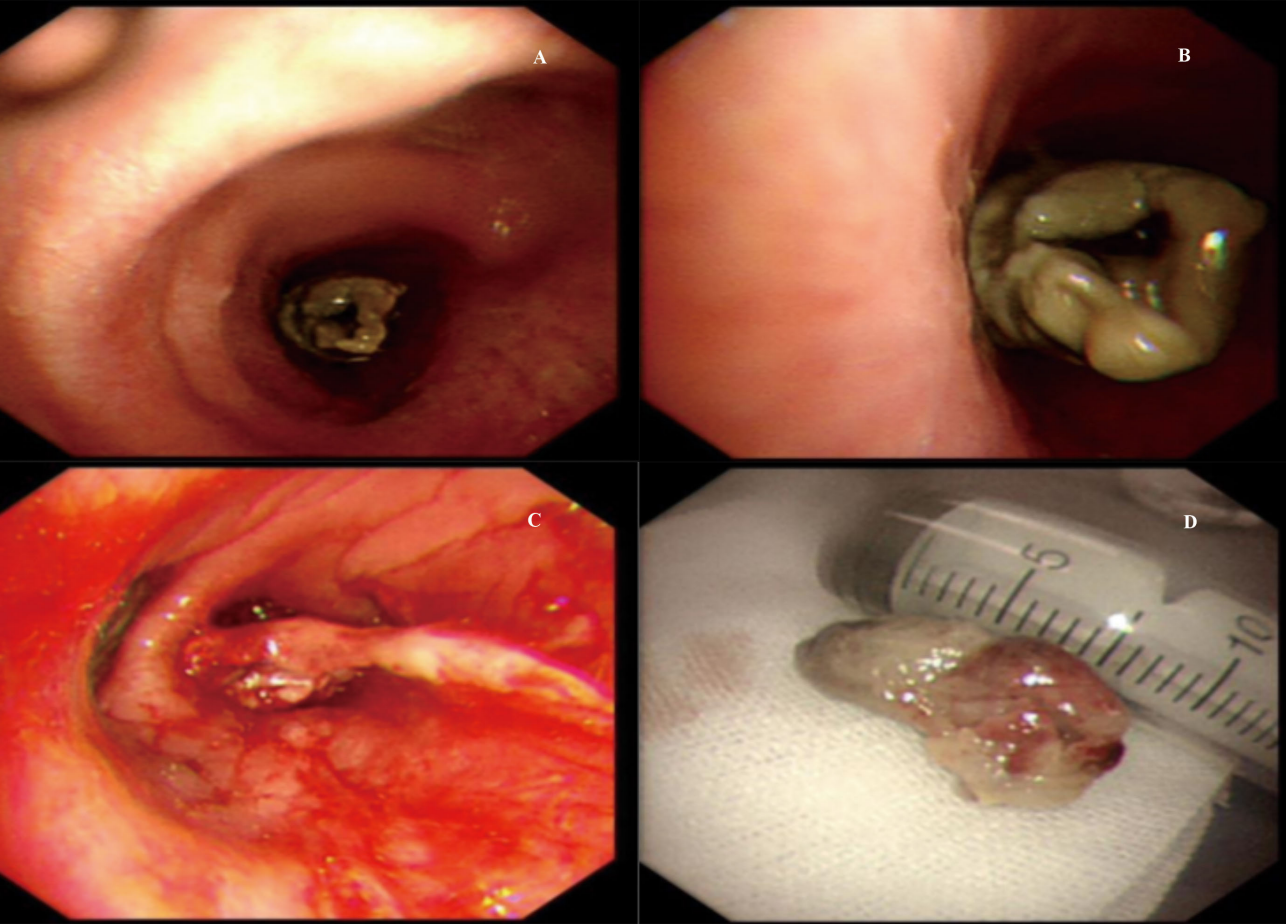
**(A–D).** Bronchoscopy reveals a grayish neoplasm blocking the lumen in the distal segment of the right main bronchus. Gross examination of the resected mass.

**Figure 4:**
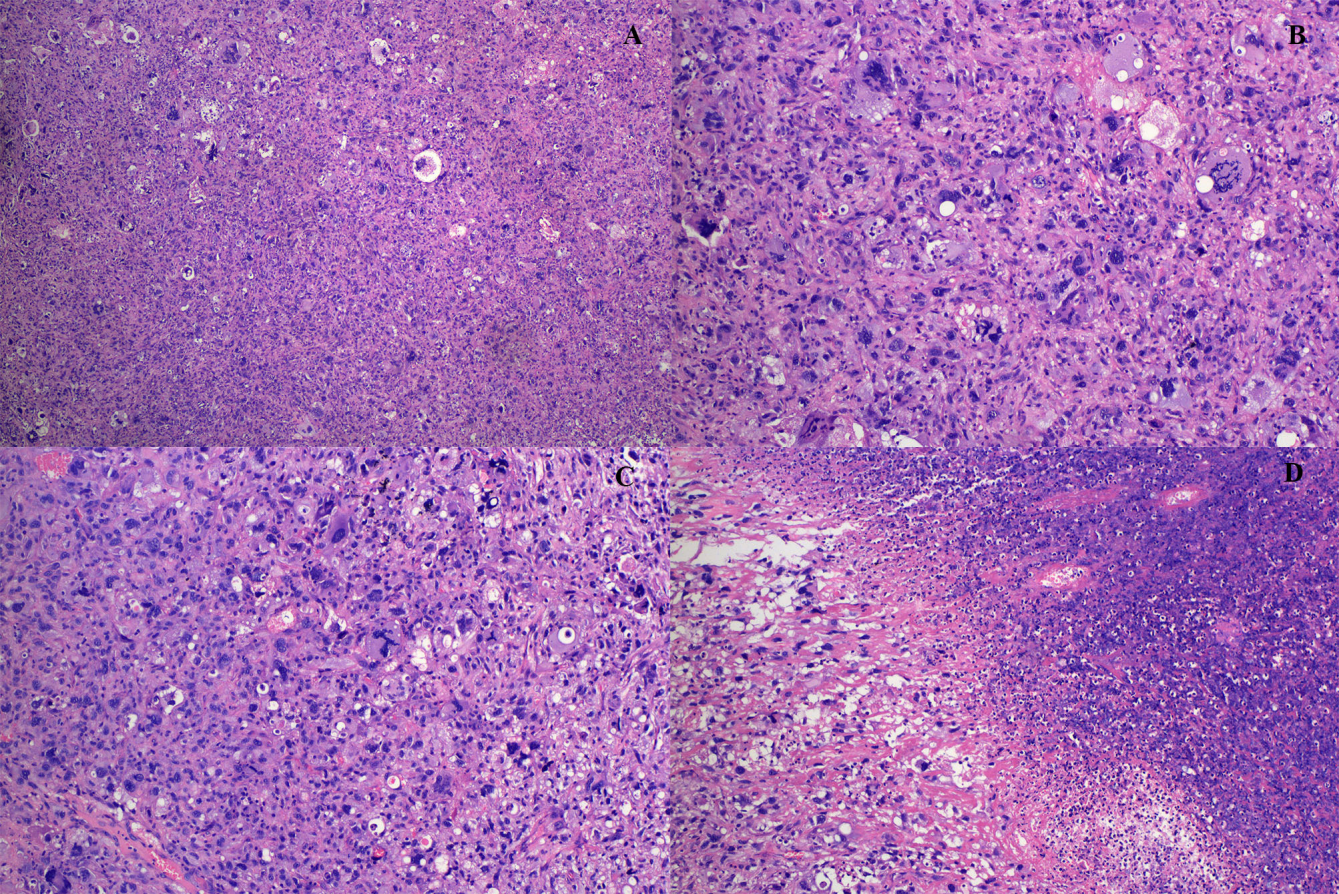
**(A–D).** Photomicrographs of the tumor showing in A**-**Vaguely nodular high-grade spindle to epithelioid neoplasm arranged in a storiform pattern (H&E, 4×); B**-**Marked pleomorphism, with variable numbers of foamy histiocytes and tumor giant cells(H&E,20×); C**-**Pleomorphic tumor cells with readily visible mitotic figures(H&E, 20×); D**-**Focal areas with extensive tumor necrosis(H&E, 20×).

**Figure 5:**
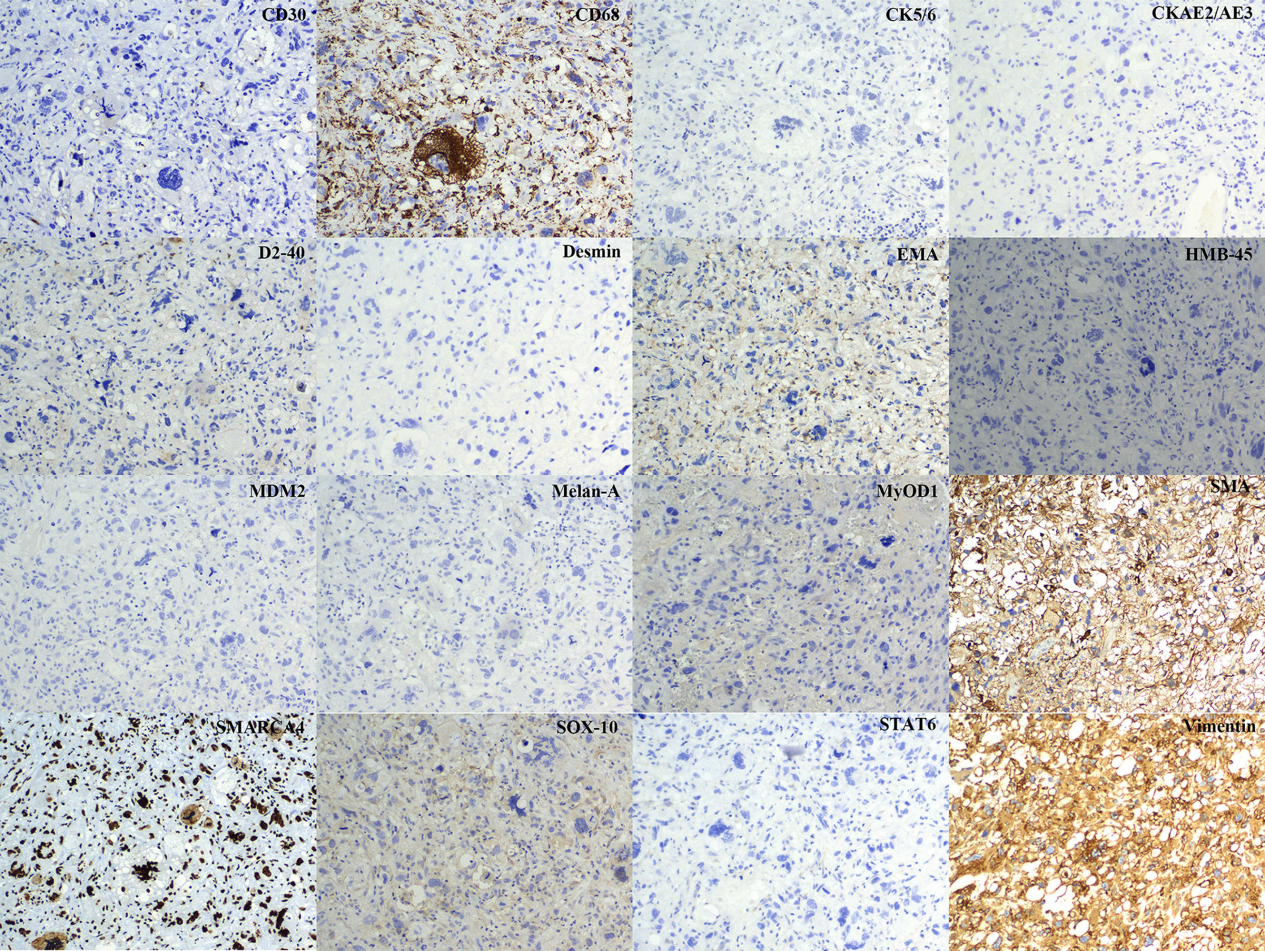
Photomicrographs of the tumor. Immunohistochemistry demonstrates diffuse positive staining for CD68 and vimentin, Immunohistochemistry studies shows no line of differentiation (Magnification ×10).

A massive solid mass (dimensions: 5.9 × 4.1 × 3.5 cm) in the right lower lobe was observed during chest contrast-enhanced computed tomography (CT). It extended along the right main bronchus and the right lower lobe bronchus (**Figures 1A–D**). The lesion showed nonuniform internal density and poorly defined borders. It displayed gradual, heterogeneous contrast enhancement on dynamic imaging. Peripheral enhancement was prominent during the arterial phase, which was further amplified during the venous phase (**Figures 2A–D**). Pulmonary function tests established that the patient’s lung capacity was drastically impaired (FEV_1_: 45% predicted, FVC: 50% predicted), which precluded curative surgical resection. A grayish mass obstructing the distal right main bronchus was detected during bronchoscopy. Owing to the patient’s impaired pulmonary function, palliative resection was carried out by employing a flexible bronchoscope and a polypectomy snare. Biopsy samples were obtained after an additional tumor tissue was observed along the bronchial wall in the right lower lobe’s basal segment (**Figures 3A–D**). The cytological examination of bronchoalveolar lavage showed a negative result for malignant cells.Representative sections revealed a vaguely nodular, high-grade spindle to epithelioid neoplasm arranged in a storiform pattern (**Figure 4A**), characterized by marked cellular pleomorphism, scattered foamy histiocytes and tumor giant cells (**Figure 4B**), conspicuous mitotic figures within pleomorphic tumor cells (**Figure 4C**), and focal extensive tumor necrosis (**Figure 4D**). Immunohistochemical staining results were as follows: NapsinA (−), TTF-1 (−), Ki-67 (+75%), Cytokeratin AE1/AE3 (−), P40 (−), Syn (focal+), Vimentin (+), Desmin (−), SMA (partial+), CD34 (−), BCL-2 (−), S-100 (partial+), HMB45 (−), SOX-10 (−), Melan-A (−), STAT6 (−), CD117 (−), ERG (−), CD45 (−), EMA (−), CK5/6 (−), CD56 (−), Smarca4 (+), Myogenin (−), MyoD1 (−), caldesmon (−), and CD68 (+) (**Figure 5**). Malignant cells were not detected by exfoliative cytology. However, focal positivity for synaptophysin and S-100 suggested the possibility of melanoma or neuroendocrine tumors. Nonetheless, the distinct morphological features of the tumor, combined with negative immunostaining for CD56, STAT6, HMB45, cytokeratin AE1/AE3, and other lineage-specific markers, supported the diagnosis of primary pulmonary undifferentiated pleomorphic sarcoma (PPUPS) after comprehensive exclusion of a wide spectrum of malignant pleomorphic neoplasms. These included sarcomatoid carcinoma, sarcomatoid mesothelioma, pleomorphic liposarcoma, pleomorphic leiomyosarcoma, pleomorphic rhabdomyosarcoma, malignant peripheral nerve sheath tumor (MPNST), malignant melanoma, solitary fibrous tumor, neuroendocrine neoplasms, synovial sarcoma, SMARCA4-deficient neoplasms, perivascular epithelioid cell tumor (PEComa), metastatic gastrointestinal stromal tumor, and anaplastic large cell lymphoma.Except for the pulmonary mass, no other pathological abnormalities were noted in abdominal ultrasonography, cranial magnetic resonance imaging, and bone emission CT. Based on comprehensive morphological and immunohistochemical analyses, the tumor was recognized as a malignant mesenchymal neoplasm with undifferentiated features. Following the exclusion of metastatic origins and confirming the lack of identifiable differentiation characteristics by immunohistochemical assay and histopathological analysis, PPUPS was finally diagnosed. Given the high surgical risk situation and the patient’s severely compromised lung capacity, the clinicians opted to avoid direct radical surgical resection; this decision was also based on a comprehensive evaluation by a multidisciplinary team comprising experts from the fields of thoracic surgery, respiratory medicine, and anesthesiology and an open discussion with the patient’s family. The endobronchial tumor was resected by palliative endoscopy with a polypectomy snare through a flexible bronchoscope. The patient achieved favorable long−term survival, remaining alive at 38 months postoperation.

## 3 Discussion

PPS accounts for <0.5% of all lung cancers (4, 5). Thus far, only 96 cases of PPUPS, a highly uncommon PPS subtype, have been documented in English literature (3, 4, 6–59) (**Table 1**). Here, by using keywords related to PPUPS/MFH combined with terms “case report,” “case series,” or “retrospective study,” three public databases (Web of Science, PubMed, and Embase) were reviewed systematically from inception to February 27, 2026. To provide a robust analysis, we included only pathologically validated PPUPS cases with complete data and excluded incomplete, duplicate, and non−English studies. Two reviewers separately extracted the data, and a third reviewer was consulted for resolving discrepancies. Excel was used for subsequent analysis. Clinical features, prognostic determinants, and survival outcomes were summarized for 56 eligible patients by using descriptive and survival analyses. Overall survival (OS) was determined by log-rank tests and Kaplan-Meier survival analysis. Statistical analysis was carried out with R program 4.2.2, and statistical significance was set at P < 0.05 (two-sided).

**Table 1:** Review of reported cases of primary pulmonary undifferentiated pleomorphic sarcoma/primary pulmonary fibrous histiocytoma.

No.	Year	Reference	Age	Sex	Location	Size (cm)	LN	Tx	Survi val (mos)	F/U	smoking history	morphology	Symptoms at the admission	M
1.	1979	Bedrossian et al. (6)	51	M	LLL/RML	3	N	L	14	DOD	smoker	nodule	Asymptomatic	NEG
2.	1979	Kern et al. (7)	53	M	RLL	8	N	L	12	DOD	U	mass	Cough	Brain
3.	1979	Kern et al. (7)	25	F	LUL	3	N	Pn,X	5	NED	U	mass	cough, low grade fever, and epigastric pain	Brain
4.	1980	Chowdhury et al. (8)	52	F	RLL	5	U	C	4	DOD	U	mass	history of lymphoma s/p XRT	U
5.	1981	Paulsen et al. (9)	53	F	LLL	4	N	L	36	DOD	nonsmoker	mass	Asymptomatic	NEG
6.	1982	Mills et al. (10)	60	F	RLL	10	N	L	18	AWD	U	mass	right shoulder blade pain, and mild increase in chronic cough compared to that in the previous year	sternum
7.	1982	Mills et al. (10)	65	M	RML/RUL	U	U	L,X	56	NED	U	mass	NA	NEG
8.	1982	Sriumpai et al. (11)	41	M	RLL	9	U	L	18	DOD	U	mass	fever,cough,chest pain	U
9.	1983	Misra et al. (12)	45	M	RLL	16	P	X	10	DOD	nonsmoker	mass	nonproductive cough, mild shortness of breath, right-sided pleuritic chest pain	cerebral cortex, left lung, kidney and periaortic lymph nodes
10.	1984	Larsen et al. (13)	75	M	RUL	2.5	N	R	10	NED	U	mass	pain of recent onset in the right chest	NEG
11.	1984	Lee et al. (14)	62	M	LLL	6	N	L	12	NED	smoker	mass	Asymptomatic	NEG
12.	1984	Lee et al. (14)	54	M	LUL	7	N	C	7	DOD	nonsmoker	mass	dull pain in the left chest, dyspnea, and a nonproductive cough	hepatic
13.	1984	Lee et al. (14)	69	M	RUL	8	N	Pn, X	8	NED	nonsmoker	mass	weight loss, cough, and hemoptysis	NEG
14.	1984	Lee et al. (14)	62	F	LLL	5	N	L,X	120	NED	smoker	mass	Asymptomatic	NEG
15.	1984	Lee et al. (14)	67	M	LUL	4	N	L	60	NED	smoker	mass	Asymptomatic	NEG
16.	1984	Lessel and Erbstösser (15)	35	F	RLL	25	U	Nt	12	DOD	nonsmoker	mass	U	U
17.	1984	Silverman and Coalson (16)	56	M	LUL	8	N	C	3	AWD	U	mass	Hoarseness, cough,weight loss	U
18.	1985	Tanino et al. (17)	75	F	LLL	5	P	Nt	5	DOD	U	mass	hemoptysis	posterior mediastinu m, and metastatic tumor in the right lung
19.	1986	Venn et al. (18)	32	F	RML/RUL	U	U	L	18	NED	nonsmoker	mass	U	NEG
20.	1986	Venn et al. (18)	62	M	RUL	8	U	L	60	NED	nonsmoker	mass	a brief history of severe pain in the upper right chest	NEG
21.	1986	Venn et al. (18)	61	F	LUL	U	U	Pn	15	DWED	smoker	mass	cough and dyspnea	NEG
22.	1986	Venn et al. (18)	62	M	LUL	U	U	X	2	AWD	smoker	mass	U	intraoral
23.	1987	Hsiu et al. (19)	71	F	RUL	5	N	L	10	NED	U	U	U	U
24.	1987	Juettner et al. (20)	68	M	LLL	20	N	Nt	12	DOD	smoker	mass	collapse and unconsciousness	NEG
25.	1987	Juettner et al. (20)	58	M	RLL	5.5	P	L	12	DOD	smoker	mass	Asymptomatic	Multifocal bone
26.	1987	Ismailer et al. (21)	12	F	RUL	9	N	L	12	AWD	U	mass	U	U
27.	1987	Yousem and Hochholzer (22)	54	F	RLL	1.7	N	L	108	NED	smoker	nodule	Dyspnea cough	NEG
28.	1987	Yousem and Hochholzer (22)	33	M	RUL	3.8	N	L	84	NED	U	mass	Hemoptysis, cough	NEG
29.	1987	Yousem and Hochholzer (22)	59	M	RLL	5.9	N	L	65	NED	U	mass	Asymptomatic	NEG
30.	1987	Yousem and Hochholzer (22)	73	F	LUL	8.5	P	Pn	36	NED	U	mass	Dyspnea, weight loss	NEG
31.	1987	Yousem and Hochholzer (22)	64	M	RUL	5	N	L,X	16	NED	U	mass	Chest pain	NEG
32.	1987	Yousem and Hochholzer (22)	42	F	LLL	3	N	L	122	NED	U	mass	Asymptomatic	NEG
33.	1987	Yousem and Hochholzer (22)	57	F	RUL	4	N	Pn	1	DNED	U	mass	Asymptomatic	NEG
34.	1987	Yousem and Hochholzer (22)	80	M	LUL	3	N	L	1	DNED	U	mass	Asymptomatic	NEG
35.	1987	Yousem and Hochholzer (22)	74	M	LUL	U	N	Nt	2	DOD	U	U	Chest pain, weight loss, bloody effusion	lung, pleural
36.	1987	Yousem and Hochholzer (22)	18	M	RLL	10	N	L	1	DOD	U	mass	Shortness of breath, cough, chest pain, weight loss	chest wall
37.	1987	Yousem and Hochholzer (22)	46	F	RUL	6	P	L,X	8	DOD	U	mass	Chest pain, cough, hemoptysis	CNS, liver
38.	1987	Yousem and Hochholzer (22)	52	F	RLL	U	N	C	9	DOD	U	U	Cough (history of lymphoma s/p XRT, chemotx)	lung, liver
39.	1987	Yousem and Hochholzer (22)	52	F	LUL	4	P	L,C, X	72	DOD	U	mass	Asymptomatic	widespread
40.	1987	Yousem and Hochholzer (22)	74	F	RUL	14	P	L	24	DOD	U	mass	cough	lung, mediastinal
41.	1987	Yousem and Hochholzer (22)	69	F	RUL	8	N	X	36	DOD	U	mass	Cough, hemoptysis, shortness of breath	mediastinal
42.	1987	Yousem and Hochholzer (22)	40	F	LLL	4	N	L,X	24	DOD	U	mass	Fever	lung, pleural, chest wall
43.	1987	Yousem and Hochholzer (22)	74	M	RML	U	N	X	8	DOD	U	U	Dyspnea, hemoptysis, cough	lung
44.	1987	Yousem and Hochholzer (22)	19	M	LUL	U	N	L,C, X	14	DOD	U	U	Cough, fever, weight loss	lung, pleura, liver, soft tissue
45.	1987	Yousem and Hochholzer (22)	63	M	LLL	7	N	Nt	14	DOD	U	mass	Fever	lung, liver
46.	1987	Yousem and Hochholzer (22)	36	M	RLL	3	N	R	12	DOD	U	mass	Asymptomatic	lung, pleura, bone, kidney, abdominal
47.	1987	Yousem and Hochholzer (22)	32	M	LLL	11	P	Pn,C ,X	3	DOD	U	mass	Chest pain	lung,liver, kidney, pancreas, brain,skin, spleen
48.	1988	Casey and Peddle (23)	21	M	RUL	3	N	L	96	NED	U	U	U	U
49.	1988	Casey and Peddle (23)	46	M	LLL	10	N	L	8	NED	U	U	U	U
50.	1988	McDonnell et al. (24)	73	F	LLL	6.5	N	L	3	DOD	smoker	mass	hemoptysis,dry cough	spleen
51.	1988	Palmer et al. (25)	62	F	RLL	U	N	L	14	DOD	U	U	U	U
52.	1989	White et al. (26)	55	M	RUL	U	U	Nt	4	DOD	smoker	U	haemoptysis	bone, adrenal
53.	1990	In et al. (27)	43	F	RLL	U	U	C,X	U	U	U	mass	Chest pain	Pulmonary artery obstruction
54.	1990	Marchán and Pérez (28)	10	F	LLL	5	N	L	U	U	U	U	U	U
55.	1993	Higashiyama et al. (29)	49	F	RLL	6	P	Pn	U	NED	U	mass	Dry cough	Lung,right iliopsoas Musle, Intraoccular
56.	1995	Kamath et al. (30)	56	M	RLL	10	U	Nt	3	DOD	U	mass	Blurred vision in the right eye	Brain
57.	1996	Gómez-Román and Val-Bernal (31)	61	M	RUL	3	U	R	9	NED	U	mass	NA	NEG
58.	1996	Halyard et al. (32)	51	F	LLL	10	N	L,X	60	NED	U	mass	Pleuritic chest pain	NEG
59.	1996	Halyard et al. (32)	77	M	RML	2.2	N	L	36	NED	U	nodule	Asymptomatic	NEG
60.	1996	Halyard et al. (32)	40	M	LLL	11	P	R	6	DOD	U	mass	Cough, pain in the left posterior rib cage, fatigue, weight loss	chest, abdomen, axilla
61.	1996	Halyard et al. (32)	57	F	LUL	7.5	U	L	1	DOD	U	mass	Left homonymous hemianopsia, headache, and ataxia	Brain
62.	1996	Shah et al. (33)	9	M	LUL	6	U	L,C, X	36	NED	U	mass	Cough, weight loss, and hemoptysis	NEG
63.	1997	Barbas et al. (34)	37	M	RML/RLL	10	N	Pn	6	DNED	smoker	mass	Cough, weight loss, and hemoptysis	NEG
64.	1997	Nistal et al. (35)	12	F	LUL	7	U	C,X	5	AWD	nonsmoker	mass	Non-productive cough, chest pain, fatigue, and weight loss	NEG
65.	2000	Herrmann et al. (36)	57	M	RUL	13	U	L	12	NED	nonsmoker	mass	Car accident/hypogly cemia	NEG
66.	2000	Fujita et al. (37)	65	F	LLL	12	U	Nt	6	DOD	U	mass	Cough, exertional dyspnea, and yellow sputum	NA
67.	2001	Nonaka et al. (38)	59	M	U	4.5	U	U	U	U	U	mass	history of lymphoma s/p XRT	NA
68.	2002	Alhadab et al. (39)	56	M	LUL/LLL	U	U	Nt	4	DOD	U	mass	Cough and shortness of breath	NEG
69.	2002	Etienne-Mastroian ni et al. (5)	47	M	U	U	U	L,X	3	NED	U	U	Asthenia, weight loss, and chest pain	NEG
70.	2003	Wang et al. (40)	86	M	LLL	15	U	Nt	2	DOD	nonsmoker	mass	Exertional dyspnea and poor appetite	NA
71.	2007	Maeda et al. (41)	62	M	LUL	4.5	P	L	24	DNED	smoker	mass	Asymptomatic	NEG
72.	2007	Rzyman et al. (42)	58	M	LUL	4	N	Pn	121	NED	U	mass	cough, fever	NEG
73.	2007	Rzyman et al. (42)	61	M	RUL	7.5	N	L	7	DNED	smoker	mass	cough	NEG
74.	2007	Rzyman et al. (42)	75	M	RUL	8	P	Pn	4	DOD	nonsmoker	mass	chest pain	NEG
75.	2007	Rzyman et al. (42)	61	F	LUL	3	N	L	2	DOD	nonsmoker	mass	hemoptysis	NEG
76.	2007	Rzyman et al. (42)	54	M	RUL	9	P	L	3	DOD	smoker	mass	chest pain, cough	NEG
77.	2008	Noh et al. (43)	58	F	RUL	5	N	L,X	5	NED	nonsmoker	mass	U	NEG
78.	2010	Tsangaridou et al. (44–48)	54	M	LUL/ LLL	U	N	Pn	168	AWD	smoker	mass	U	lung,right
79.	2010	Maitani et al. (45)	18	F	LUL	2.2	U	L	36	NED	U	nodule	Asymptomatic	iliopsoas musle, Intraoccular NEG
80.	2012	Jeon and Park (46)	55	M	LLL	U	N	Pn,C ,X	9	NED	U	mass	Cough and chest pain	NEG
81.	2013	Jung-Hyun Kim MD (47)	61	M	REB	3	N	E	36	NED	smoker	nodule	shortness of breath and chest discomfort	NEG
82.	2013	Li et al. (48)	80	F	RUL	8	U	Nt	1.5	DOD	U	mass	Cough	NA
83.	2013	Thomas and Koshi (49)	47	M	RUL	U	P	C,X	2	DOD	nonsmoker	mass	Swelling on the gingiva	liver,bone
84.	2015	Patel et al.3	86	M	RLL	9.6	N	L	6	NED	U	mass	Cough, increasing weakness, and dyspnea	NEG
85.	2017	Li et al. (50)	61	M	RUL	8	P	L	U	U	smoker	mass	intermittent cough with blood expectoration	NEG
86.	2017	Tuğba Coşgun (51)	50	M	LUL	10	N	L	36	NED	smoker	mass	dyspnea and chronic cough	NEG
87.	019	Amir Qorbania (4)	66	M	RLL	15	N	L,C	4	NED	U	mass	weight loss (7 kg), right-sided chest wall pain, night sweats, and fatigue	NEG
88.	2019	Miyashita (52)	71	M	RML	3	N	L	15	NED	smoker	mass	cough	NEG
89.	2019	Sanja Pleština (53)	57	M	LUL	10	N	L	2	DOD	smoker	mass	cough and hemoptysis	peritoneal ,s mall bowel
90.	2020	Higuchi M (54)	52	F	LUL	15	N	L	24	NED	U	mass	dyspnea and severe cough	NEG
91	2020	Zhen Xu (55)	45	F	LLL	7	N	LC,X	U	U	nonsmoker	mass	long-term persistent cough and blood-containing sputum	Renal and sacrum
91.	2021	Kilitci A (56)	52	F	RLL	U	U	C	4	DOD	U	mass	cough and shortness of breath	NEG
92.	2021	Do Kyun Kang (57)	56	M	RUL	7	N	C	U	U	smoker	mass	U	NEG
93.	2022	Shuai Zhang (58)	59	M	RML/RLL	5.7	P	T	19	DOD	smoker	Mass	Cough, chest tightness, hemoptysis, headache, dizziness, and shortness of breath	Brain and bone
93.	2023	Rajpoot A (59)	49	M	R	23	N	Nt	U	DOD	U	mass	chest pain, fatigue, and weight loss	widespread
94.	2025	Present case	78	M	REB	3	N	E	38	NED	smoker	nodule	cough and hemoptysis	NEG

AWD, alive with disease; C, chemotherapy; DNED, dead with no evidence of disease; DOD, death due to disease; DWED, death due to unrelated cause with evidence of disease; E, endoscopic resection; F, female; F/U, follow-up; L, lobectomy; LN, lymph node; LLL, left lower lobe; LUL, left upper lobe; M, male; Nt, no treatment; NED, no evidence of disease; NEG, negative; Pn, pneumonectomy; POS, positive; R, resection; REB, right endobronchial; RLL, right lower lobe; RML, right middle lobe; RUL, right upper lobe; T, targeted immunotherapy; Tx, treatment; U, unavailable data; X, radiotherapy.

**Figure 6:**
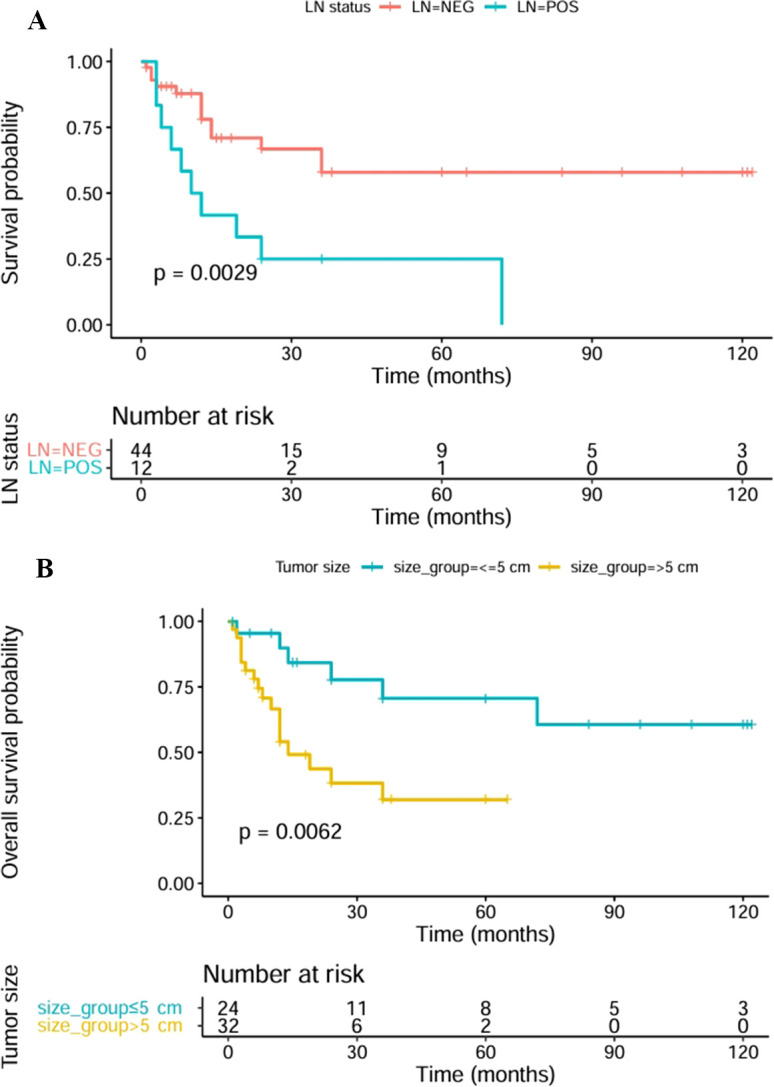
The 5-year OS rates showed significant differences between the no lymph node metastatic and lymph node metastatic groups (blue and red lines, 55.3% and 25%, respectively; p = 0.0029) **(A)**. The 5-year OS rates showed significant differences in the tumor size ≤ 5 cm and tumor size >5 cm groups (blue and yellow lines, 62.5% and 41.9%, respectively; p = 0.0062) **(B)**.

The study group comprised 63.6% males and 36.4% females; the mean age was 55.9 ± 18.5 years (range: 9–86 years). The predominant condition was right lung involvement (55.4%), while bilateral involvement was rare (1.8%). The median tumor size was 7.0 cm (interquartile range: 4.0–10.0 cm). Lymph node metastasis was noted in 21.4% patients, while nodal negativity correlated with considerably improved OS (**Figure 6A**). Tumor size of ≤5 cm emerged as a favorable prognostic factor (**Figure 6B**) (12). Furthermore, 66.1% patients underwent surgical resection alone, resulting in a 5-year OS rate of 52.0% within the initial cohort of 56 patients (the dataset of 56 patients exhibited comparable trends). The OS rates at 1, 3, and 5 years were 66.3%, 50.9%, and 50.9%, respectively, with 12.0 months as the median OS period. This observation corroborated earlier data (50, 52) and highlighted the aggressive property of PPUPS and the importance of optimized treatment strategies. Notably, only two endobronchial PPUPS cases were identified in this cohort, and both these patients had good outcomes. In certain high-risk patients, long-term local control of lesions confined to the bronchus can be achieved through minimally invasive endobronchial resection; this confirms earlier findings that intrabronchial lesions show better prognosis than lesions at other sites (9–47) and highlights the significance of detecting resectable lesions promptly (3). The multivariate analysis showed strong influence of total surgical resection on patient OS, while nodal status serves as an independent prognostic factor. Complete resection is the mainstay of curative treatment; careful dissection of lymph nodes can facilitate precise staging and inform adjuvant therapy-related decisions (**Table 2**).

**Table 2:** Patients’ characteristics (n = 56).

Characteristic	Statistic
Age (years)	
Mean ± SD	55.9 ± 18.5
Sex	
Male	36 (64.3%)
Female	20 (35.7%)
Tumor size (cm)	
Median (IQR)	7.0 (4.0 – 10.0)
Treatment modality	
Surgery alone	37 (66.1%)
Surgery + Radiotherapy	8 (14.3%)
Surgery + Chemotherapy	1 (1.8%)
Surgery + Chemotherapy + Radiotherapy	2 (3.6%)
Chemotherapy alone	1 (1.8%)
Radiotherapy alone	2 (3.6%)
No treatment	2 (3.6%)
Tumor location (by lobe)	
RUL	15 (26.8%)
RLL	10 (17.9%)
RML	2 (3.6%)
REB	2 (3.6%)
LUL	12 (21.4%)
LLL	12 (21.4%)
Multiple lobes	3 (5.4%)
Tumor location (by side)	
Right	31 (55.4%)
Left	24 (42.9%)
Bilateral	1 (1.8%)
Overall survival rates *	
1-year	66.3%
3-year	50.9%
5-year	50.9%
Lymph node metastasis	
Positive	12 (21.4%)
Negative	44 (78.6%)

This analysis reveals several important findings: (1) PPUPS is more common in men and exhibits a specific inclination for the right lung; additionally, a large tumor size and nodal metastasis are adverse prognostic factors; (2) complete tumor resection is a critical determinant of cure; timely detection of endobronchial lesions or small lesions (≤5 cm) optimizes the survival of patients; and (3) compared to single-modality treatment, multimodal strategies or minimally invasive endobronchial resection (for endobronchial lesions) may offer improved clinical outcomes for high-risk patients. This study supports current evidence regarding PPUPS, confirming its aggressive behavior, crucial prognostic factors (nodal status and total resection), and favorable outcomes for rare endobronchial cases. Prompt detection, comprehensive staging, and tailored surgical approach, potentially utilizing minimally invasive approaches where appropriate, could maximize patient’s survival; future investigations should be aimed at multimodal therapy enhancement.

While middle-aged to elderly males are the primary population affected by PPUPS, very few cases in children have also been documented (3, 32). At the preoperative stage, PPUPS is often misdiagnosed due to its indistinct clinical symptoms, ranging from asymptomatic presentation to cough, chest pain, or dyspnea (3). On radiological examination, PPUPS most often appears as a single peripheral mass characterized by clear edges, intense contrast enhancement, and heterogeneous density due to hemorrhage or necrosis (5, 57, 60, 61). In the current case, the tumor displayed a very unusual growth as an endobronchial lesion along the right main bronchus, which is rare and can be easily confused with primary bronchogenic carcinoma. Consistent with earlier reports (62, 63), the progressively increasing enhancement pattern of the tumor indicates its hypervascular nature. Radiologists should consider PPUPS when performing differential diagnosis for endobronchial masses exhibiting aggressive behavior. Recognizing the primary imaging features might help differentiate PPUPS from other complications. The contrast enhancement pattern is a critical characteristic: remarkable homogeneous hyperenhancement (≥30 HU) is observed for typical carcinoid tumors, while malignant epithelial tumors (e.g., adenoid cystic carcinoma [ACC] and squamous cell carcinoma [SCC]) show mild-to-moderate enhancement with rapid washout (64). Peripheral enhancement of PPUPS and other bronchial sarcomas exhibit typical variations, which stem from their mesenchymal origin; in contrast, benign lesions (e.g., hamartoma and chondroma) generally display mild and homogenous enhancement (64, 65). Regarding tumor shape and margins, benign lesions such as papillomas and polyps are tiny with clear borders (65). Malignant epithelial tumors, however, tend to have irregular edges, possibly resulting in secondary obstructive changes. Additionally, SCC presents as ulcerative mural thickening of the lower trachea (enhancement >40 HU) and is strongly correlated with smoking (64, 66), and ACC depicts longitudinal tumor growth (>3 cm) accompanied by circumferential lumina narrowing (64, 67). Bronchial sarcomas, such as chondrosarcoma and PPUPS, generally present as huge, highly invasive, and infiltrative masses that have poorly defined borders; they often extend beyond the trachea (79% of cases) and may exhibit varying levels of calcification (up to 71% of chondrosarcomas) (64). Additional auxiliary diagnostic clues include lymphadenopathy (highly suggestive of malignancy), calcification (observed in carcinoid [30% of cases], chondroma, and tuberculosis) (64, 65), and necrosis (common in malignant neoplasms). The CT-based differential diagnosis of bronchial sarcomas remains difficult primarily due to their extreme rarity (mainly described as isolated cases or incidental discoveries), histopathological heterogeneity, and overlapping imaging features with other tracheobronchial neoplasms. Because of these radiographic ambiguities, definitive diagnosis must be made through histopathological confirmation by conducting detailed morphological and immunohistochemical assessments (68, 69). In the retrospective analysis of the 56 cases from the literature, PPUPS predominantly presents as a solitary pulmonary parenchymal mass (48/56, 85.7%), defined by the presence of nonuniform boundaries, heterogeneous density, and sporadic occurrence of cavitation/calcification. Conversely, endobronchial lesions without parenchymal involvement are a rare incidence (2/56, 3.6%). The present case is a rare endobronchial PPUPS. Chest CT showed a lower lobe segmental endobronchial lesion (smooth borders, homogeneous density, and absence of parenchymal invasion/cavitation/calcification). Similar to most endobronchial mesenchymal tumors, the tumor in the present case showed irregular contours and heterogeneous internal density. The lesion exhibited progressive and heterogeneous enhancement after dynamic contrast-enhanced imaging, marked by notable peripheral enhancement in the arterial phase and additional augmentation in the venous phase. These imaging features provide valuable insights into differential diagnosis.

Pulmonary UPS originates from primitive mesenchymal cells that lack definitive differentiation or specific immunohistochemical markers (70, 71). Macroscopically, these tumors appear as sizable, firm masses with hazy boundaries, grayish-white to reddish-gray cut surfaces, and the frequent existence of necrosis/hemorrhage (72, 73). Microscopically, UPS is defined by prominent cellular pleomorphism, atypia, disorganized arrangement of variable tumor cells, multinucleated giant cells, interstitial collagen deposition, foamy xanthoma cells, inflammatory infiltrates, high mitotic activity, prominent angiogenesis, and extensive collagenous stroma (74, 75). Our patient showed very high Ki-67 proliferation index (75%), reflecting aggressive tumor growth and its association with unfavorable prognosis. This finding corroborates with the malignant characteristic of this rare sarcoma subtype (76, 77). UPS exhibits substantial morphological pleomorphism with an indeterminate lineage of differentiation. Consequently, establishing a definitive diagnosis requires excluding a wide range of malignant pleomorphic neoplasms, including sarcomatoid carcinoma, sarcomatoid mesothelioma, pleomorphic liposarcoma, pleomorphic leiomyosarcoma, pleomorphic rhabdomyosarcoma, malignant peripheral nerve sheath tumor (MPNST), malignant melanoma, solitary fibrous tumor, neuroendocrine tumors, synovial sarcoma, SMARCA4-deficient tumor, perivascular epithelioid cell tumor (PEComa), metastatic gastrointestinal stromal tumor, and anaplastic large cell lymphoma. Given these entities’ overlapping morphological features and their marked pleomorphism, coupled with existence of pleomorphic multinucleated giant cells and poor cellular differentiation, a comprehensive diagnostic approach integrating extensive sampling, molecular pathological analysis, and immunohistochemical assay is imperative for precise differential diagnosis. Immunohistochemical analysis and molecular studies can help exclude other tumors with identical histological findings (**Table 3**). UPS typically expresses histiocytic markers (CD68 and vimentin) and lacks lineage-specific markers (4, 78). In the present case, tumor cells demonstrated diffuse and robust positivity for CD68 and vimentin, while displaying negativity for epithelial markers such as cytokeratin AE1/AE3, p40, TTF−1, EMA, p63, cytokeratin 5/6, and NapsinA, effectively ruling out epithelial-derived malignant neoplasms such as sarcomatoid carcinoma and epithelioid sarcoma (78–80); additionally, positive SMARCA4 staining was identified, which excluded the possibility of SMARCA4-deficient malignant tumors, as SMARCA4 deficiency constitutes their defining characteristic (81, 82). A negative result for high CD30 expression led to the exclusion of anaplastic large cell lymphoma (83, 84). Malignant melanoma, a key mimic exhibiting diffuse pleomorphism, was excluded due to the negative result for melanocytic markers (HMB-45, Melan-A, and SOX10) (81, 85). CD34 (-) and CD117 (-) ruled out metastatic gastrointestinal stromal tumor (GIST), as CD117 is a core marker of GIST and CD34 is often co-expressed (86). SOX10 (-) excluded MPNST, as SOX10 is a specific marker for neural crest-derived cells (87, 88). STAT6 (-) ruled out solitary fibrous tumor, given its characteristic nuclear expression in this tumor (80, 89). Neuroendocrine tumors were excluded based on CD56 (-) and focal weak positivity for synaptophysin (Syn); isolated weak Syn expression without the presence of other neuroendocrine markers is insufficient for confirmation (90, 91). HMB45 (-) and desmin (-) excluded PEComa, as HMB45 is specific and desmin is occasionally expressed in PEComa (92). CD34 (-) and ERG (-) eliminated angiosarcoma (ERG is specific for vascular endothelial cells) (93). CK5/6 (-), BCL (-), and EMA (-) ruled out synovial sarcoma, as these markers are frequently associated with their epithelioid component (94, 95). Additionally, although SMA was partially positive (SMA, partial +), the absence of myogenic markers myogenin (-), MyoD1 (-), and caldesmon (-) essentially excluded leiomyosarcoma (96–98). MDM2 (-), CDK4 (-), and no abnormal fusion genes on genetic testing excluded dedifferentiated liposarcoma (MDM2/CDK4 amplification and fusion genes are the key indicators) (99). Tumor cells displayed negative results for skeletal muscle lineage markers (desmin, myogenin, and MyoD1), which further excluded the diagnosis of pleomorphic rhabdomyosarcoma (100, 101). Moreover, WT1 (-), CK5/6 (-), and D2-40 (-) facilitated the differential diagnosis of sarcomatoid mesothelioma, and their combined negative expression effectively eliminated this entity (102). The distinct immunophenotype and substantial pleomorphism eventually confirmed the diagnosis of UPS. Notably, the majority of patients with PPUPS displayed extrapulmonary metastases, highlighting the critical need for careful clinical evaluation to facilitate precise diagnosis.

**Table 3:** Ancillary tests for differentiating tumors exhibiting sarcomatoid characteristics.

Tumor	Immunohistochemical outcomes	Molecular profile
Sarcomatoid Carcinoma	P40+, P63+, TTF1+, pankeratin+, epithelial markers (MUC31, EMA, Ber-EP4, monoclonal CEA, B72.3)	Gains at chromosomes 8q, 1q, 3q, 19p; KRAS mutation; loss of function of CDKN2A at 9p21 on IHC; EGFR mutation
Sarcomatoid Mesothelioma	CytokeratinAE1/AE3+,CK5/6+, WT1+, calretinin+, D2-40+	Inactivation of BAP1, EZH2 at 1q21 or FISH for CDKN2A deletion
Synovial Sarcoma	TLE1+, Keratin+, S100+/-,EMA+, BCL-2+	t(X;18) involving SS18 (SYT) gene
Epithelioid Sarcoma (ES)	MUC4+, CD34-, EMA+, keratin+, vimentin+	SMARCB1 (INI1) gene alterations on IHC (22q11)
Dedifferentiated Liposarcoma	CDK4+, MDM2+, desmin-, S100-, SMA+	Ring and giant marker chromosomes derived from 12q13-15 (amplification of MDM2, SAS, CDK4, HMGA2)
Anaplastic large-cell lymphoma	CD45+, CD30+, ALK+/-	ALK gene rearrangement
Melanoma	SOX10+, S100+, MITF+, MelanA/MART1+, HMB45+, tyrosinase+	BRAF, NRAS, GNAQ, BAP1, KIT, HRAS, NF1, TERT, and PTEN mutations
Malignant peripheral nerve sheath tumor	GFAP+, S100+, vimentin+, CD34+	Complex
Solitary fibrous tumor	STAT6+, CD34+, BCL2+	NAB2-STAT6 fusion
Leiomyosarcoma	desmin+, SMA+, vimentin+, caldesmon+	Complex
Rhabdomyosarcoma	desmin+, myogenin+, MyoD1+	MYC (3q28) or FLT4 (5q33) amplification, amplification of vascular endothelial growth factor receptor (VEGFR, KDR, TIE1, TEK)
Angiosarcoma	Vascular markers (CD31, ERG, FLI1, CD34)+, keratin +/- in epithelioid angiosarcoma	MYC (8q24) or FLT4 (VEGFR3) (5q35) amplification, upregulated vascular-specific receptor tyrosine kinases (KDR, TIE1, FLT1, TEK)
SMARCA4-deficient undifferentiated tumor	SMARCA4-,SMARCA 2-,SMARCB1(INI1)+,CK7+/-,vimentin +,Claudin-4 +/-,TTF-1-,P63-,EMA-, stem cell markers(CD34, SALL4, SOX2)+	Deletion of SMARCA4 genes
Perivascular epithelioid cell tumor	SMA+, desmin+, HMB45+, MITF+, MART1+	TSC2 mutations, TFE3 gene fusions

As a very uncommon subtype of UPS/MFH, PPUPS usually exhibits aggressive biological behavior, high local recurrence and metastasis rates (13–42% and 31–35%, respectively), and minimal response to conventional treatments (103). A carefully tailored management strategy is required for PPUPS because of poor outcomes following tumor metastasis and the moderate extent of the 5-year disease-specific survival rate (63%) for UPS patients; this strategy should be driven by insights provided by a multidisciplinary team due to the unavailability of standardized treatment protocols (103). Although radical surgery is usually considered the main curative strategy for localized UPS (104), our patient’s case indicates that a more tailored approach should be adopted for high-risk individuals, particularly when conventional open surgery might cause major complications or pose a high risk. In patients with advanced UPS, systemic chemotherapy using anthracyclines, such as doxorubicin (with or without ifosfamide), shows modest effectiveness (27–33% response) (105); moreover, this treatment’s significance as an adjuvant in localized PPUPS remains controversial, as the current state of evidence does not convincingly demonstrate the survival benefits of this approach (61, 106). Likewise, while adjuvant radiotherapy (RT) reduces local recurrence likelihood, it also exposes patients to the risk of developing radiation-associated secondary sarcomas—a subset with worse outcomes and distinct molecular profiles (elevated phosphorylated/total IGF-1R) compared to sporadic lesions (103, 107). Regarding molecular traits, LAPTM4A/B gene-regulated chemoresistance pathways and epithelial-mesenchymal transition contribute to enhance PPUPS’s aggressive nature (61, 73), thereby further complicating systemic management. A high Ki-67 index (exceeding 50%) is a known unfavorable prognostic factor of PPUPS and shows a correlation with a greater risk of recurrence and metastasis and a shorter survival period (103, 104). Interestingly, after undergoing minimally invasive endobronchial resection, our patient had a 38-month disease-free survival (DFS) period, despite displaying a high Ki-67 index. This outcome is probably because of the distinct confinement of the tumor in the endobronchial space (without distant metastasis or parenchymal invasion during tumor diagnosis) and full bronchoscopic resection. This observation demonstrates that in select PPUPS patients with localized lesions, complete minimally invasive resection can counteract the poor prognostic impact of high tumor proliferation. This result agrees with the limited evidence supporting the adoption of organ-preserving, minimally invasive strategies to prioritize quality of life while still achieving good oncological outcomes for specific PPUPS cases (108). In addition to surgical approaches, recently discovered therapies provide more options for treating PPUPS. Targeted agents such as PI3K/mTOR inhibitors (ridaforolimus) (109), multi-kinase inhibitors (cabozantinib and sunitinib) (110, 111), and anti-AXL agents (for UPS with AXL overexpression) (103) show potential, while alectinib has been used to successfully target rare actionable alterations (e.g., pulmonary UPS displaying EML4-ALK rearrangement), yielding an OS period of 19 months (112). Presently, immunotherapy is considered a transformative strategy for eliminating these tumors: pembrolizumab monotherapy achieves a 40% objective response rate in patients with advanced UPS, with superior benefits in PD-L1+ (≥5%) or high-tumor mutational burden (>5 mutations/Mb) patients; likewise, perioperative pembrolizumab combined with surgery and RT improves 2-year DFS by 15% in patients with stage III extremity UPS by leveraging RT-induced formation of tertiary lymphoid structure (TLS) and T cell infiltration (113, 114). To lower chemoresistance or prevent immunological desert phenotype, various combination therapies are currently under investigation, such as immune checkpoint inhibitors (ICIs) with adenosine pathway inhibitors or M2 macrophage-targeting inhibitors (112). Multifaceted prognostic factors influence UPS tumors: positive outcomes are associated with favorable clinical variables (for example, tumor < 10 cm, age < 61 years, negative lymph node metastasis, and margin-negative resection) (103, 114), while tumor microenvironment features (such as high CD3+/CD8+ T cell count or infiltration with CD68+/CD163+ macrophages) predict better survival outcomes for UPS patients but not for myxofibrosarcoma patients, highlighting subtype-specific differences (111). Prognostic classification and customized treatment can be improved by molecular indicators such as PTEN loss, AXL overexpression, activation of the RAS/MAPK pathway, and ctDNA dynamics (54, 103, 114). Although there have been remarkable advancements in treating UPS, several problems remain unresolved. These include treatment response-related heterogeneity, restricted accessibility, and ICI resistance mechanisms, which necessitate biomarker-specific evaluations, innovative combination therapies, and establishment of subtype-based protocols according to the data from longitudinal multi-omics analysis. A transcriptomic profiling study found three UPS subgroups with differing outcomes, suggesting individualized risk stratification (7). Prognostic evaluation for PPUPS requires integrating clinical factors (tumor ≥7 cm, age ≥60 years, lymphovascular invasion, and lymph node involvement), molecular factors (overexpression of AXL, loss of PTEN, and Ki-67 index), and immunological factors (103, 104). Current concerns in this domain include inconsistent use of multimodal therapy, retrospective bias, and scant PPUPS-related data. Prognosis improvement for this very rare, aggressive sarcoma may primarily rely on subtype-specific treatment customized based on the distinct molecular profile of UPS, together with greater adoption of minimally invasive resection for certain localized cases.

Compared to most previous single-center case series, the present study has several strengths, such as strict patient selection criteria to guarantee data completeness, a comparatively large cohort size (n = 56), and comprehensive survival analyses to recognize prognostic markers. However, due to the study’s retrospective design, there are some limitations, including selection bias, lack of standardized adjuvant treatment protocols during study, and a short follow-up duration for certain patients. Additional multicentric prospective studies with a greater sample size are needed to confirm the current findings, establish the optimal multimodal treatment strategies, and develop novel prognostic biomarkers or therapeutic targets for PPUPS.

In summary, PPUPS is a highly uncommon mesenchymal tumor lacking distinct clinical signs and radiological features. Chest CT can detect peripheral soft tissue masses with reasonably clear borders, heterogeneous density, limited spiculation, central necrosis or cystic alterations, and marked enhancement of solid components on contrast-enhanced scans. These features, when observed in chest CT findings, should prompt PPUPS consideration in differential diagnosis. Extrathoracic metastases can be ruled out by using positron emission tomography-CT. Total surgical resection remains the primary therapeutic approach, potentially followed by adjuvant chemotherapy and/or RT to enhance therapeutic results. This case emphasizes two crucial points: (1) PPUPS can occur as a single endobronchial mass and (2) patients ineligible for radical surgery may benefit from minimally invasive local therapy. Hence, molecular characterization and optimal multimodal strategies should be addressed in future studies for treating PPUPS.

## Data Availability

The original contributions presented in the study are included in the article/supplementary material. Further inquiries can be directed to the corresponding author.
